# Ophthalmology

**Published:** 1907-09

**Authors:** A. Ogilvy


### OPHTHALMOLOGY.

At a recent meeting of the Ophthalmological Society held in Edinburgh,[2] Sydney Stephenson read a most instructive paper on the subject of the " spirochæta pallida in relation to the **syphilitic affections of the eye.** He prefers to call the organism of Schaudinn by its newer name of treponema pallidum, which organism has now been found in all forms and stages of syphilis, whether acquired or inherited, human or animal. From the ophthalmic point of view the following facts may be stated : (1) The treponema has been found in the apparently healthy eyes of fœtuses and of infants who have died from congenital syphilis ; (2) It has been found in lesions set up experimentally in the eyes of animals by the inoculation of syphilitic material, such as chancres or diseased glands ; (3) It has been found in actual syphilitic invasions of the human eye. The conclusion is inevitable, that in the discovery of Schaudinn's organism we have the strongest possible proof of the syphilitic nature of any given disease of the eye. A point concerning interstitial keratitis may be cleared up by our present knowledge, which has long been a mystery. The

[1] *Ann. Surg.*, 1907, i. 203. [2] *Ophthalmoscope*, June 1st, 1907.

existence of spirochætes in the cornea, iris and choroid of the syphilitic infant gives us a hint as to how interstitial keratitis-iritis and choroiditis may be brought about later on in life. Although most of the protozooa may succumb to the natural defensive powers of the body, yet some may remain latent, perhaps in a resting stage, like the trypanosome, until roused into renewed life and activity by an exciting cause, possibly years after the original invasion. On this theory it becomes comparatively easy to understand why in predisposed subjects local injury is so commonly an exciting cause of interstitial keratitis, and why a history of traumatism is far from unknown in choroiditis in those who have acquired a hereditary syphilis. The persistence of a few spirochætes, whilst most had undergone destruction, would explain the relapses now and then observed in cases of interstitial keratitis, and similar considerations would account for the recurrences, not infrequent, of syphilitic choroiditis. In relation to this, it may interest the members of the Society to remember a case of interstitial keratitis with disseminated choroiditis recently shown at the Society's clinical evening. I mentioned the frequent occurrence of injury as an immediate cause of the specific inflammatory attack, and quoted three cases, two of brothers who had normal eyes till over twenty, when one developed keratitis following an injury from dust blowing in his eye, the other one keratitis, from a blow with a hammer in the eye. In each of these case the keratitis came on in the injured eye, but quickly spread to the uninjured organ. The third case was a man who went on apparently with good eyes till the age of 35, when a blow from a twig set up an irritation which ended in a definite attack of interstitial keratitis.

With interstitial keratitis on one's mind, its exceedingly slow course and the difficulty of finding a remedy naturally occurs to one. Mercury and potassium iodide, although no doubt both excellent in their way, seem to take less effect in interstitial keratitis than in any of the other eye troubles due to syphilis. Iritis and choroiditis both yield to their influence as to a charm, but keratitis distinctly hangs fire. Latterly dionine has come into vogue, and in dionine a good friend has been found. Combined in an ointment with atropin, in the proportion of dionine 2 per cent., atropin 1 per cent., an excellent result may be obtained. The application in the form of ointment introduced between the lids seems to take much more effect than when used as drops. Possibly the ointment, adhering to the conjunctiva for a longer time than the drops remain there, may have something to do with it; but be that as it may, dionine and atropin ointment have for me worked wonders when all other remedies have failed miserably. I can recall a young lady, whose interstitial keratitis, a very definite attack too, got well in exactly three weeks. I had prepared her, as I always do, for a long course of treatment, and mentioned three months as a likely period. This is my own experience. Listen then to the experience of others. Karl Grossmann, of Liverpool, says[1] of dionine, "yet it is a drug which, if once used, with discrimination and without prejudice, is not likely to be omitted from the oculist's outfit. Though a derivate of morphia, it is characterised by the comparative absence of its toxicity, which makes it a safe remedy in eye practice. When brought into contact with the conjunctiva, either in solution, ointment, or in substance, a burning sensation is soon followed by lachrymation and fine injection, often combined with reduced sensibility ; on the ocular conjunctiva a fine cobweb of lymphatic vessels becomes visible, and is gradually swallowed up by a more or less strongly-developed chemosis, which often covers the corneal limbus by a sausage-like swelling. The lids swell to such an extent that they cannot be opened, and this state reaches its maximum in about half an hour, when the œdema gradually lessens, to disappear in three to six or even twelve hours."

The action of dionine on the eye is a powerful lymphagogue ; it acts, however, as an analgesic as well, but not as an anæsthetic, like cocain. In iritis and iridocyclitis, Grossmann considers we come to the affections in which dionine appears to do most good, and here the analgesic effect on the deep-seated pain is most remarkable. Every now and then one comes on a case where dionine does not produce its usual chemotic action, and in these cases it does least good ; furthermore, eyes soon become used to dionine, and after the fourth or fifth application the chemosis is almost *nil*. As soon as this takes place its good effect also disappears, and increasing the dose, even to dusting the eye with the actual powder itself, produces but little reaction ; its use should, therefore, be discontinued for several days, when once more its beneficial results will be apparent.

Thompson Henderson has written an excellent article on the **healing of the corneal wound in cataract extraction.**[2] This paper is based on the examination of thirty-three eyes which had been operated on for cataract. In all these the clinical process of healing was progressing, or had progressed in a perfectly normal manner. In twenty-one of the cases death supervened from some intercurrent affection, in a period varying from three days to a month after the date of operation ; in four the eyes were enucleated for pain and secondary glaucoma, while eight specimens were obtained from the *post-mortem* room. This gives a very good range to judge the healing processes by. He points out that in an experimental corneal incision there is a greater retraction of the anterior and posterior than of the central corneal layers, so

[1] *Folia Therap.*, 1907, i. 53. [2] *Ophth. Rev.*, May, 1907.

that the margins of the wound, instead of appearing as two straight lines, show curved surfaces, meeting and touching in the middle, giving the appearance of two triangular spaces with bases respectively in and out. In cataract extraction wounds this appearance, while present, is modified and not evident, on account of the nature and position of the incision. Irregularities of the opposing surfaces are not uncommon, in consequence of the sawing movements with which sections are often completed, giving the wound track a notched, wavy or step-like appearance. He divides the process of repair into three stages: mediate union, primary union, permanent union and cicatrisation, the last of which, he points out, takes a much longer time to accomplish than is generally thought.

Mediate union is brought about by a fibrinous exudate, which glues the margins of the wound together at that point where the distance between them is least. This fibrinous plug is sufficient to retain the aqueous and allow of the restoration of the anterior chamber. He thinks the fibrinous plug is chiefly, if not altogether, a derivative of the altered aqueous humour, as in non-perforating wounds of the cornea, the cut surfaces show little or no fibrinous deposit.

The primary union stage is effected by the surface epithelium and the posterior endothelium. These layers proliferate and grow down the respective margins of the incision till they meet and cover not only the lips of the wound, but also the fibrinous plug that brought about the mediate union. This stage takes very varying times in its accomplishment, from three days to even a fortnight, or even longer after the operation. This disparity is ascribed to the personal factor of the vital activity of the tissues in different cases. This epithelial growth introduces one of the dangers following extraction, if the wound remains in a gaping condition, or the fibrinous plug is not strong enough to resist the growth of epithelium. This latter grows down and over the edge of the corneal wound, and may eventually progress over the posterior surface of the cornea on to the surface of the iris, and so block the corneo-iritic angle, causing glaucoma, for which two of the eyes were eventually enucleated—four and five years respectively—after what at first had been looked on as most successful operations.

While the stratified epithelium on the surface of the cornea is descending into the outer part of the wound, the endothelium on the posterior surface by a similar process lines the inner aspect of the incision to the completion of this primary union. Henderson points out that primary union in a corneal flap incision is thus brought about and completed without the parenchyma playing any part in the process; and judging from the series of cases examined, it is certainly not till the sixteenth day that the corneal elements proper manifest any active sign of reparative activity, that is, not before the average patient in this country is discharged from hospital.

Permanent union is brought about by a slow and gradual growth of the corneal fibres; these, by their pressure on the epithelial plug, cause it to atrophy and finally to disappear entirely. The interspace between the two cut surfaces is thus reduced to a vanishing point, so that the normal radius of curvature of the cornea is restored.

Firm and permanent cicatrisation is not accomplished for two, three or more months, but when completed it is the exclusive product of the corneal parenchyma, with a course which it is scarcely possible to follow in its entirety. This length of time in regeneration explains a point with which we were often struck in cases of discission for congenital cataract, viz. the ease with which it is possible to reopen a paracentesis wound, even as long as three weeks after it had been first made, with an ordinary repositor, by making quite a gentle pressure on the upper edge of the corneal wound.

A. OGILVY.

